# Supplementary material for: CD169+ Macrophages in Primary Breast Tumors Associate with Tertiary Lymphoid Structures, Tregs and a Worse Prognosis for Patients with Advanced Breast Cancer
Source: Cancers (Basel). 2023 Feb 16;15(4):1262. doi: 10.3390/cancers15041262 (PMC9954705; doi:10.3390/cancers15041262)

Table S1: Odds ratio table comparing CD169$^+$/TLLS$^+$ co-infiltration and TLLS$^+$ infiltration with tumor and metastasis clinicopathological features and other immune cell infiltration.

**Clinicopathological features**		**CD169$^+$/TLLS$^+$ PT**				**CD169$^+$/TLLS$^+$ LNM**				**TLLS$^+$ PT**				**TLLS$^+$ LNM**			
		**OR**	**95% CI**	**P-value[a]**	**N**	**OR**	**95% CI**	**P-value[a]**	**N**	**OR**	**95% CI**	**P-value[a]**	**N**	**OR**	**95% CI**	**P-value[a]**	**N**
Age	*>50 y*	1			180	1			74	1			85	1			48
	<50 y	0.65	0.192 – 2.214	0.54	11	0.64	0.295 – 1.383	0.25[b]	41	1.97	0.81 – 4.78	0.14[b]	106	0.84	0.39 – 1.79	0.70[b]	67
Overall Survival	*>5 y*	1			180	1			74	1			67	1			42
	<5 y	**0.29**	0.080 – 1.014	**0.053**	11	**3.51**	1.429 – 8.603	**0.005**[b]	41	**0.41**	0.18 - 0.94	**0.045**[b]	124	**2.63**	1.20 – 5.76	**0.018**[b]	73
Relapse free interval	*>5 y*	1			170	1			71	1			108	1			77
	<5 y	0.31	0.065 – 1.478	0.20	11	1.75	0.773 – 3.971	0.18[b]	41	**0.31**	0.11 - 0.85	**0.018**[b]	73	1.64	0.70 – 3.81	0.30[b]	35
Tumor Size	*T1*	1			179	1			74	1			80	1			34
	>T1	1.29	0.365 – 4.569	0.76	11	0.48	0.211 – 1.101	0.081[b]	40	1.19	0.51 – 2.78	0.83[b]	110	0.62	0.27 – 1.43	0.30[b]	80
Ki67 PT	*Low*	1			167	1			66	1			115	1			75
	High	**5.43**	1.386 – 21.276	**0.018**	11	1.09	0.448-2.635	0.85[b]	38	2.04	0.881-4.725	0.092[b]	63	0.94	0.395-2.258	0.90[b]	29
Ki67 LNM	*Low*	1			87	1			64	1			62	1			69
	High	3.33	0.526 – 21.114	0.33	5	0.57	0.210-1.530	0.26[b]	29	2.90	0.718-11.712	0.122[b]	30	1.30	0.530-3.169	0.57[b]	30
ER PT	*Neg*	1			172	1			68	1			36	1			21
	Pos	**0.26**	0.076-0.920	**0.042**	11	1.51	0.532-4.285	0.44[b]	38	0.49	0.19 - 1.24	0.18[b]	147	0.92	0.35 – 2.46	0.87	85
ER LNM	*Neg*	1			89	1			67	1			24	1			28
	Pos	**0.10**	0.010-1.043	**0.051**	4	0.50	0.119-1.241	0.13[b]	31	**0.17**	0.04 - 0.79	**0.025**[b]	69	0.48	0.19 – 1.22	0.12[b]	70
PR PT	*Neg*	1			171	1			65	1			79	1			42
	Pos	0.92	0.269-3.116	1	11	0.92	0.407-2.067	0.83[b]	38	0.51	0.22 - 1.18	0.14[b]	103	0.66	0.30 – 1.47	0.32[b]	61
PR LNM	*Neg*	1			89	1			66	1			57	1			60
	Pos	0.37	0.040-3.429	0.65	5	0.56	0.222-1.388	0.21[b]	31	0.63	0.15 - 2.61	0.74[b]	37	**0.43**	0.18 – 0.99	**0.044**[b]	37
HER2 PT	*Neg*	1			171	1			171	1			166	1			95
	Pos	2.49	0.490 – 12.68	0.25	11	2.49	0.490 – 12.678	0.25	11	2.18	0.65 – 7.37	0.25	16	0.92	0.24 – 3.47	1.0	10
HER2 LNM	*Neg*	1			79	1			79	1			69	1			77
	Pos	2.79	0.234 – 33.264	0.41	3	2.79	0.234 – 33.264	0.41	3	2.96	0.48 - 18.12	0.24	12	0.71	0.21 – 2.41	0.76	12
**Cell infiltration association**																	
FoxP3 PT	*Neg*	1			164	1			60	1			68	1			40
	Pos	**0.59**	0.515- 0.666	**0.007**	11	1.45	0.619-3.384	0.39[b]	36	**8.54**	1.94 - 37.63	**0.001**[b]	107	1.91	0.83 – 4.41	0.13[b]	56
FoxP3 LNM	*Neg*	1			70	1			61	1			24	1			26
	Pos	0.66	0.555- 0.778	0.55	3	1.47	0.466-4.641	0.59	19	2.61	0.29 - 23.71	0.66	49	1.83	0.71 – 4.72	0.21[b]	54
CD169 PT	*Neg*	1			180	1			65	1			138	1			79
	Pos	---	---	---	11	2.43	0.514 – 11.505	0.25[b]	38	**3.77**	1.61 - 8.82	**0.004**[b]	53	1.02	0.40 – 2.62	0.97[b]	24
CD169 LNM	*Neg*	1			96	1			74	1			50	1			57
	Pos	2.5	0.462 – 13.521	0.44	7	---	---	---	41	2.04	0.58 – 7.27	0.36[b]	53	**4.76**	2.12 – 10.71	**0.0001**[b]	58

Abbreviations: PT = Primary Tumor. LNM = Lymph node metastases. OR = Odds ratio. 95% CI = 95% confidence interval. N = Number of patients. ER = Estrogen Receptor. PR = progesterone Receptor. HER2 = Human epidermal growth factor receptor 2.

a= Fisher exact test unless otherwise stated. two tailed

b= Pearson's chi square test. two-tailed

Supplementary Figure legends

Figure S1:

Kaplan Meier survival showing the differences in 25 year breast cancer specific survival (BCSS) and recurrence free interval (RFI) for specific immune cell populations infiltrating lymph node metastases (LNM). *P* values by the log rank test are highlighted in bold when significant. On the left, the impact of immune cell infiltration for $CD169^+$ TAMs, TLLS and $CD169^+$ TAMS/TLLS was investigated as prognostic markers for BCSS. On the right, the impact of $CD169^+$ TAMs, TLLS and $CD169^+$ TAMS/TLLS was investigated as a prognostic marker for RFI. Green lines indicate LNMs with $CD169^+$ TAMs, TLLS or $CD169^+$ TAMS/TLLS infiltration and black lines indicate the absence of $CD169^+$ TAMs, TLLS or $CD169^+$ TAMS/TLLS.

Figure S1

Lymph Node Metastases
Long term prognosis

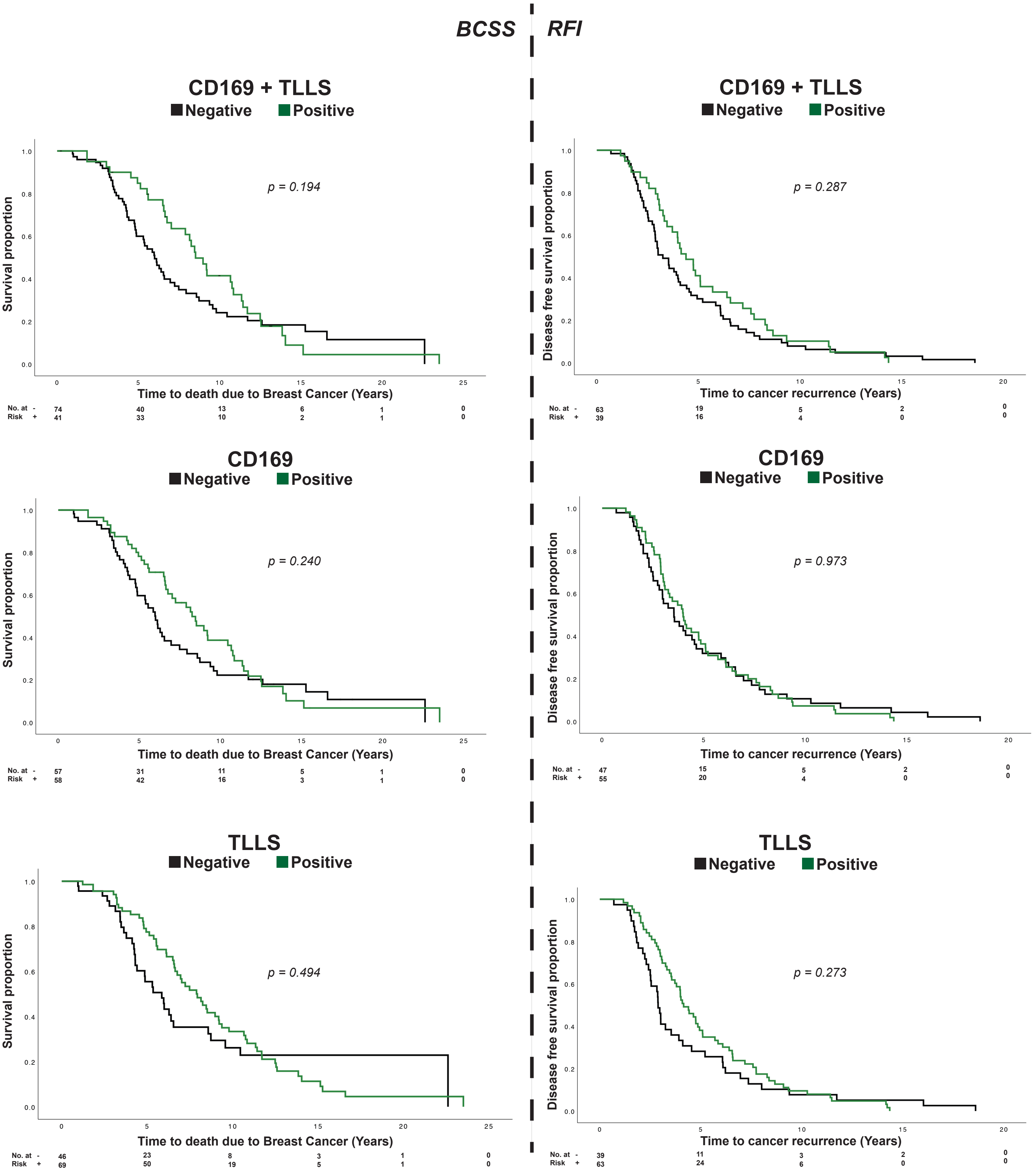

Figure S2:

Kaplan Meier analyses performed on matched donors. Four types of associations were compared: patients lacking immune cells infiltration in both PT and LNM, patients having immune cells infiltrating in both PT and LNM and patients having cell infiltration in either PT or LNM. In the left panel, BCSS was investigated for all three types of cell infiltration. In the right panel, the same analysis was performed for RFI. *P* value by the log rank test.

Figure S2

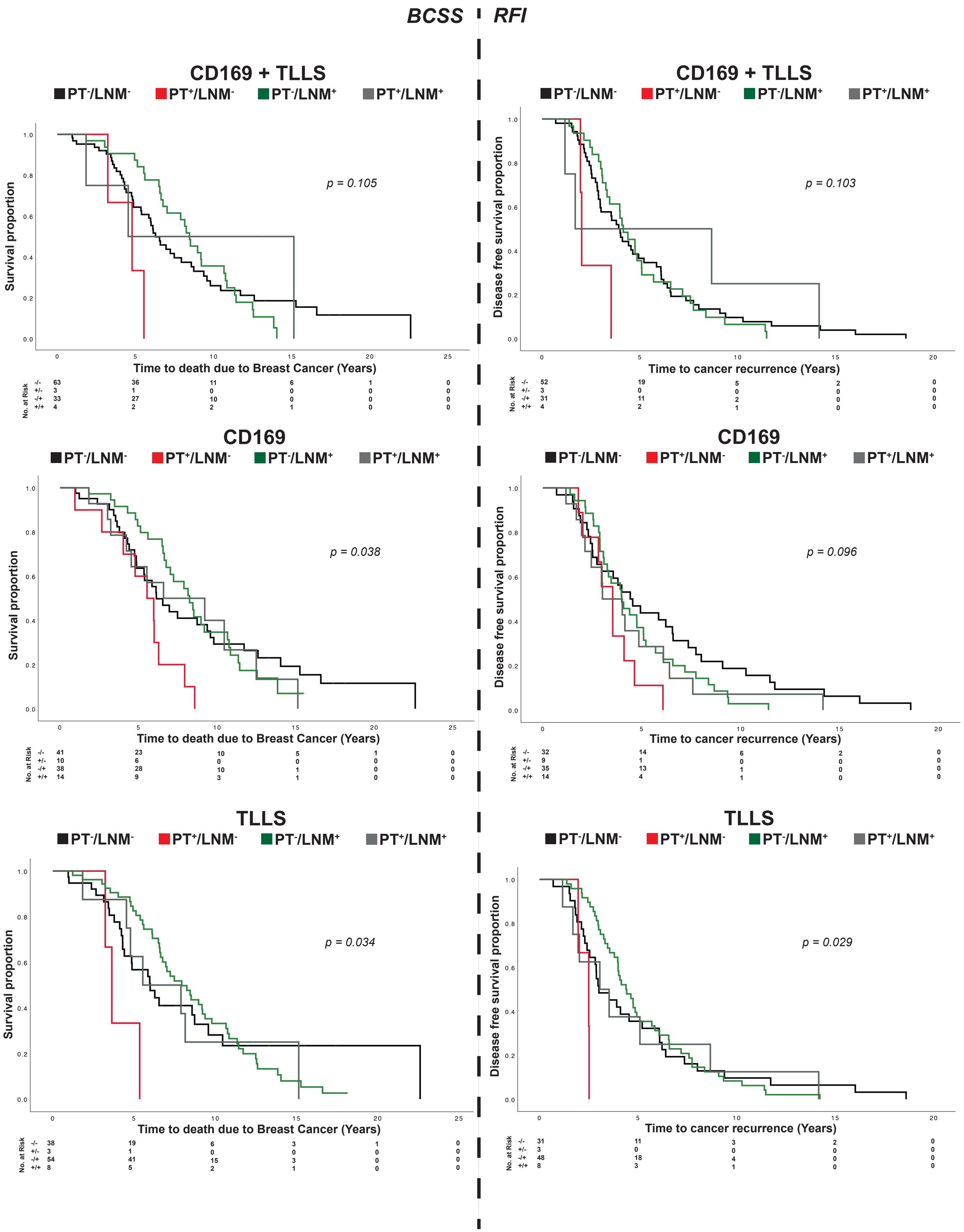

BCSS
RFI
CD169 + TLLS
PT-/LNM-
PT+/LNM-
PT-/LNM+
PT+/LNM+
Survival proportion
p = 0.105
Time to death due to Breast Cancer (Years)
No. at Risk
-/- 63 36 11 6 1 0
+/- 3 1 0 0 0 0
-/+ 33 27 10 0 0 0
+/+ 4 2 2 1 0 0
CD169 + TLLS
Disease free survival proportion
p = 0.103
Time to cancer recurrence (Years)
No. at Risk
-/- 52 19 5 2 0
+/- 3 0 0 0 0
-/+ 31 11 2 0 0
+/+ 4 2 1 0 0
CD169
Survival proportion
p = 0.038
Time to death due to Breast Cancer (Years)
No. at Risk
-/- 41 23 10 5 1 0
+/- 10 6 0 0 0 0
-/+ 38 28 10 1 0 0
+/+ 14 9 3 1 0 0
CD169
Disease free survival proportion
p = 0.096
Time to cancer recurrence (Years)
No. at Risk
-/- 32 14 6 2 0
+/- 9 1 0 0 0
-/+ 35 13 1 0 0
+/+ 14 4 1 0 0
TLLS
Survival proportion
p = 0.034
Time to death due to Breast Cancer (Years)
No. at Risk
-/- 38 19 6 3 1 0
+/- 3 1 0 0 0 0
-/+ 54 41 15 3 0 0
+/+ 8 5 2 1 0 0
TLLS
Disease free survival proportion
p = 0.029
Time to cancer recurrence (Years)
No. at Risk
-/- 31 11 3 2 0
+/- 3 0 0 0 0
-/+ 48 18 4 0 0
+/+ 8 3 1 0 0

Figure S3:

Forest plots with Cox regression analyses (BCSS). Forest plots showing Cox regression analysis on 25 year BCSS (PT) and 10 year BCSS (LNM) in breast cancer patients with $CD169^+$ TAMs, TLLS or $CD169^+$ TAMS/TLLS presence, adjusted individually and all together for confounders such as receptor expression status (ER, PR, HER2), T_{regs} presence, Ki67 levels, tumor size and age at diagnosis. Hazard ratios are indicated with dots together with horizontal lines representing the 95% confidence interval.

Figure S3

Multiple Cox regression analysis
Forest Plots - Breast cancer specific survival

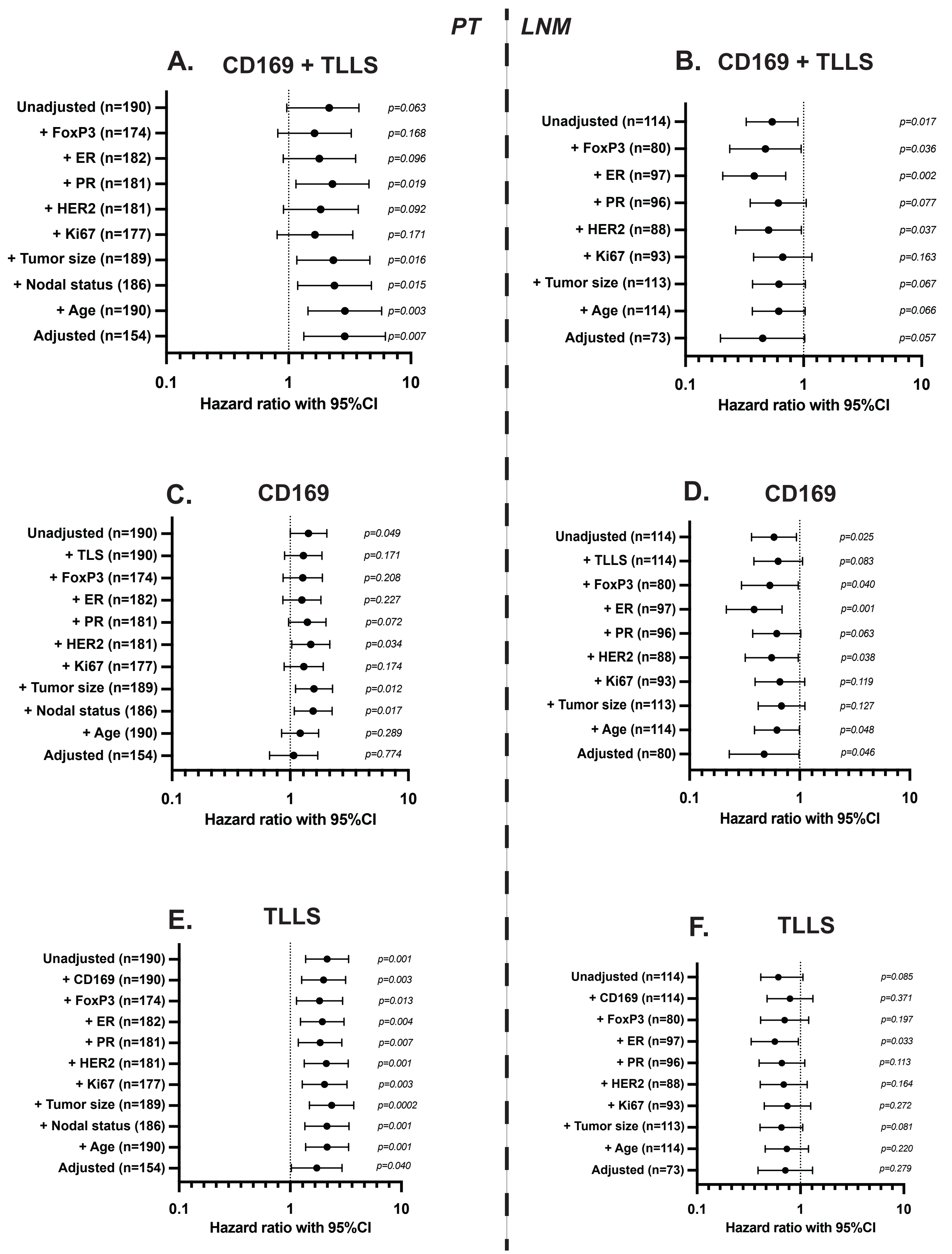

Figure S4:

Forest plots with Cox regression analyses (RFI). Forest plots showing Cox regression analysis on 25 year RFI (PT) and 10 year RFI (LNM) in breast cancer patients with $CD169^+$ TAMs, TLLS or $CD169^+$ TAMS/TLLS presence, adjusted individually and all together for confounders such as receptor expression status (ER, PR, HER2), T_{regs} presence, Ki67 levels, tumor size and age at diagnosis. Hazard ratios are indicated with dots together with horizontal lines representing the 95% confidence interval.

Figure S4

Multiple Cox regression analysis

Forest Plots - Recurrence free interval

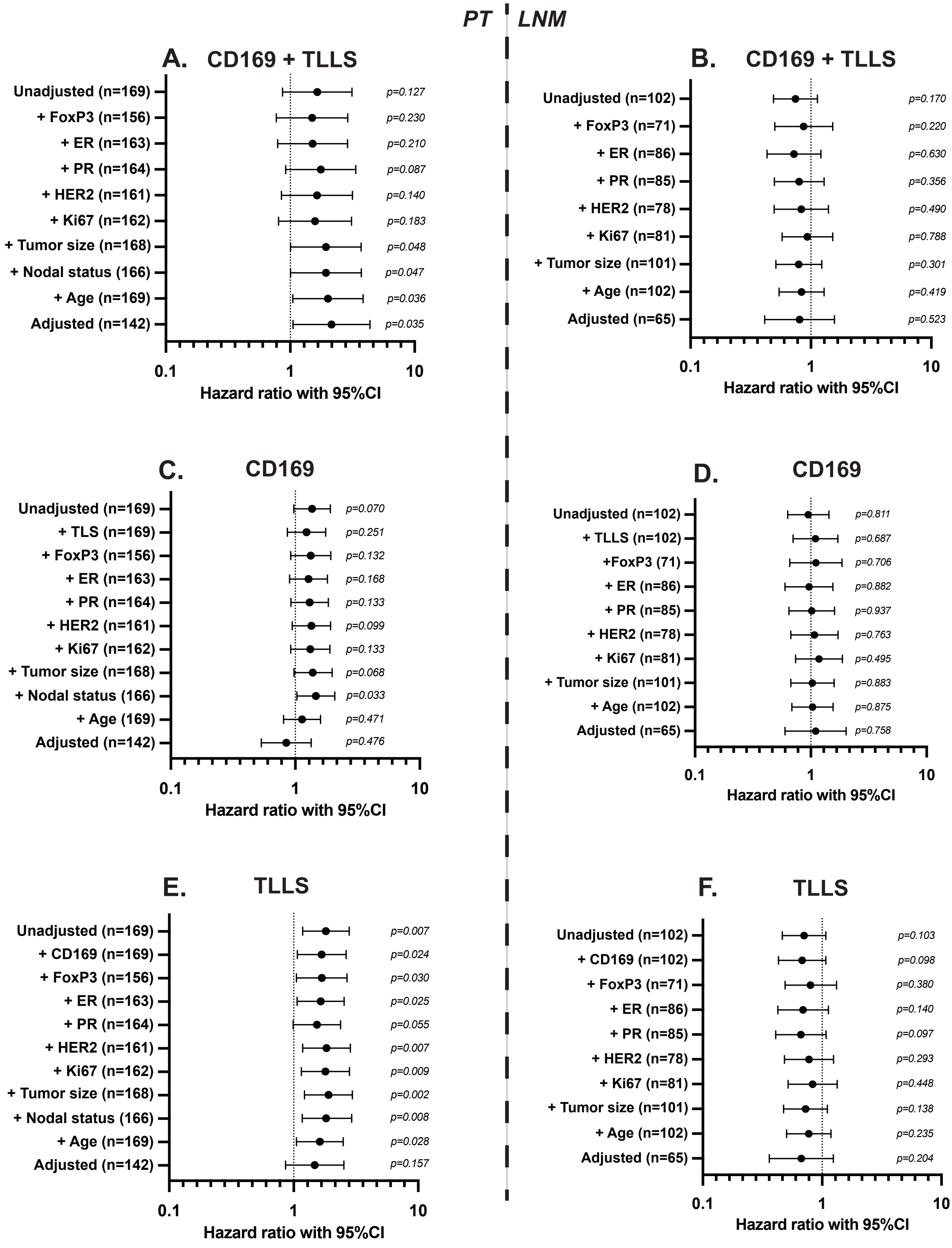

Figure S5:

Complete heat maps, containing sample hierarchical clusters with Pearson correlations between *SIGLEC1* (CD169) and gene signatures for tertiary lymphoid structures (TLS), B_{regs} or T_{regs}. Patients are characterized based on their molecular subtype of breast cancer, aggressive subtypes in red (Basal-like, $HER2^{+}$ and Luminal B) and luminal A or normal-like subtypes in green. Upregulated genes are shown in yellow while downregulated genes are shown in blue. The threshold for upregulation/downregulation was set at 1/-1 based on median centered gene relations. The highlighted areas represent clusters with positive cell infiltration correlations shown in Figure 4.

Figure S5

Complete sample Hierarchical clustering with pearson correlations

Downregulated genes *Upregulated genes*

Basal
Her2
Luminal B

Normal-like
Luminal A

CD169 gene signature in relation to TLS gene signature, Median centered genes relations

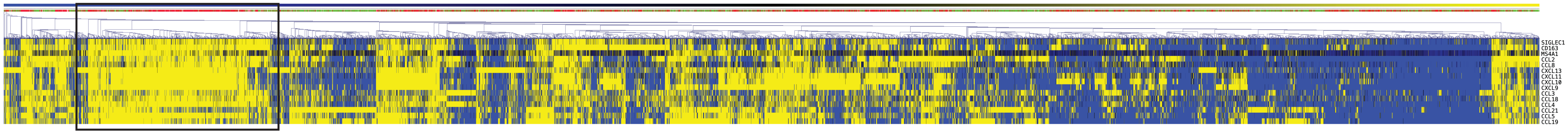

CD169 compared to Breg gene signature, Median centered genes relations

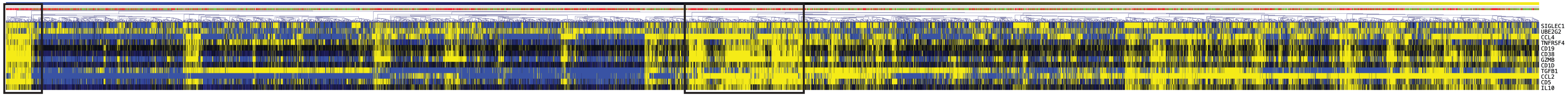

CD169 compared to TLS, Breg and Treg gene signatures, Median centered genes relations

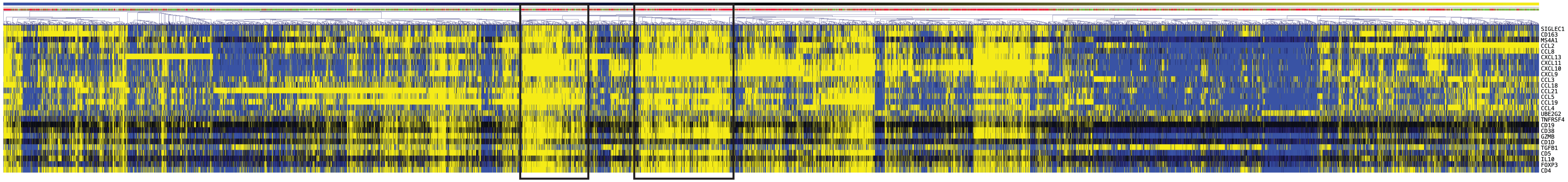

CD169 differential gene signature, Median centered genes relations

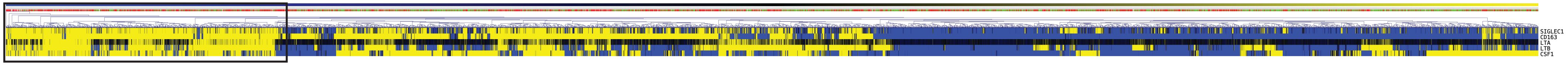

Supplement: Supplementary file 1 [file cancers-15-01262-s001.zip › cancers-2208184-supplementary.pdf]
